# Antibiotic Resistance Pattern and Evaluation of Metallo-Beta Lactamase Genes Including *bla*-_*IMP*_ and* bla*-_*VIM*_ Types in *Pseudomonas aeruginosa* Isolated from Patients in Tehran Hospitals

**DOI:** 10.1155/2014/941507

**Published:** 2014-04-23

**Authors:** Samira Aghamiri, Nour Amirmozafari, Jalil Fallah Mehrabadi, Babak Fouladtan, Hossein Samadi Kafil

**Affiliations:** ^1^Department of Microbiology, Islamic Azad University, Lahijan Branch, Lahijan, Iran; ^2^School of Medicine, Microbiology Department, Iran University of Medical Sciences, Tehran, Iran; ^3^Department of Bioscience and Biotechnology, Malek-Ashtar University of Technology, Tehran, Iran; ^4^Drug Applied Research Center, Tabriz University of Medical Sciences, 5166614766 Tabriz, Iran

## Abstract

Beta-lactamase producing strains of *Pseudomonas aeruginosa* are important etiological agents of hospital infections. Carbapenems are among the most effective antibiotics used against *Pseudomonas* infections, but they can be rendered infective by group B **β**-lactamase, commonly called metallo-beta lactamase. In this study, the antimicrobial sensitivity patterns of *P. aeruginosa* strains isolated from 9 different hospitals in Tehran, Iran, as well as the prevalence of MBLs genes (*bla*-_*VIM*_ and *bla*-_*IMP*_) were determined. A total of 212 strains of *P. aeruginosa* recovered from patients in hospitals in Tehran were confirmed by both biochemical methods and PCR. Their antimicrobial sensitivity patterns were determined by Kirby-Bauer disk diffusion method. Following MIC determination, imipenem resistant strains were selected by DDST method which was followed by PCR tests for determination of MBLs genes: *bla*-_*IMP*_ and *bla*-_*VIM*_. The results indicated that, in the DDST phenotypic method, among the 100 imipenem resistant isolates, 75 strains were MBLs positive. The PCR test indicated that 70 strains (33%) carried *bla*-_*VIM*_ gene and 20 strains (9%) harbored *bla*-_*IMP*_. The results indicated that the extent of antibiotic resistance among *Pseudomonas aeruginosa* is on the rise. This may be due to production of MBLs enzymes. Therefore, determination of antibiotic sensitivity patterns and MBLs production by these bacteria, can be important in control of clinical *Pseudomonas* infection.

## 1. Introduction

*Pseudomonas aeruginosa *is one of the commonest causes of infection in burn patients and an important agent for hospital acquired infections and death in immunocompromised such as cystic fibrosis and cancer patients [1]. This bacterium is often resistant to many antimicrobial agents. The cause of resistance can be efflux pumps, decreased outer membrane permeability, and secretion of beta-lactamase enzymes [2]. Several kinds of beta-lactamase enzymes have been recognized. These enzymes were initially seen in Gram-negative bacteria which were detected in periplasmic space [3]. Metallo-beta lactamases are classified in group B of Ambler classification. This group is divided into three subclasses: BI, BII, and BIII. The BI subclass is divided into four categories according to their molecular structures: the IMP, VIM, GIM, and SPM types [4]. The first MBLs enzymes were IMP-1 which was initially found in* S. marcescens* in Japan (1991), VIM-1 originally detected in Italy (1997), SPM-1 first detected in Brazil (1997), and finally GIM detected in Germany (2002) [5, 6]. Carbapenems are effective antibiotics against* Pseudomonas* infections. But because the genes for MBLs are often carried on plasmids and class I integron, they can rapidly spread among different species of this bacterium and other bacteria [7, 8]. MBLs can potently hydrolyze all beta-lactam antibiotics except azetreonam. These enzymes require zinc ion as cofactor [9]. Hence, their activity is inhibited by chelators like ethylenediaminetetraacetic acid (EDTA), sodium mercaptoacetic acid (SMA), 2-mercaptopropionic acid (MPA), and dipicolinic acid (DPA). Sulbactam, tazobactam, and clavulanic acid which are often used to inhibit beta-lactamase enzymes are not effective against MBLs [2, 10]. Several phenotypic methods are available for detection of MBLs producing bacteria. All these methods are based on the ability of metal chelators such as EDTA to inhibit the activity of MBLs. The double disk synergy test method was employed in this investigation [9]. The goal of this study was to determine the antibiotic resistance pattern in* P. aeruginosa *species isolated from nine hospitals in Tehran, Iran, and evaluate the prevalence of MBLs genes,* bla-*_*VIM*_ and* bla-*_*IMP*_, in imipenem resistance strains.

## 2. Materials and Methods

### 2.1. Collection of Strains

A total of 212 strains of* P. aeruginosa *were collected during six-month period from October 2011 to March 2012 from Motahari, Shariati, Hashemi Nejad, Kasra, Hazrat Rasoul, Milad, Mehr, Tebbi Kodakan, and Baghiatallah hospitals in Tehran, Iran. These strains were isolated from wound, blood, urine, trachea, sputum, pleural fluid, eye, catheter, and larynges samples. 148 isolates were obtained from male patients and 64 isolates from female patients. These isolates were subcultured on Brucella agar and their identification was performed by both biochemical methods such as oxidase test, catalase test, OF test, growth at 42°C, and PCR using specific primers for* oprL* gene (*oprL* is a constitutively produced peptidoglycan-associated lipoprotein which contains covalently bound fatty acyl chains) [11]. Bacterial strains were preserved in Trypticase soy broth.

### 2.2. Antibiotic Susceptibility Tests

Antimicrobial sensitivity tests were performed on Mueller-Hinton agar (Biolab-Hungary) by Kirby-Bauer disk diffusion method [12] and interpreted according to CLSI (Clinical and Laboratory Standards Institute) standard tables.* Pseudomonas aeruginosa* ATCC_27853_ was used as control for the susceptibility tests. The antibiotic disks used were Imipenem (10 *μ*g), Ciprofloxacin (5 *μ*g), Gentamicin (10 *μ*g), Tetracyclin (30 *μ*g), Ceftazidime (30 *μ*g), Cefotaxime (30 *μ*g), Azithromycin (15 *μ*g), Tobramycin (10 *μ*g), Ticarcylin (75 *μ*g), and Piperacillin (100 *μ*g) (Padtan Teb, Iran). At first the bacteria were cultured into TSB and incubated at 35°C for 24 hours. After 24 hours, microbial suspension was prepared equivalent to the turbidity of 0.5 McFarland standard. With sterile swabs they were plated on MH agar. The antibiotic disks were placed on the plate and incubated at 35°C for 24 hours. Following incubation, the diameters of the zone of inhibition were measured.

### 2.3. Minimum Inhibitory Concentration (MIC)

Determination of MIC was performed for imipenem resistant strains by agar dilution method according to CLSI standards. Isolates with MIC value of ≥16 *μ*g/mL were screened as MBLs producing strains.* Pseudomonas aeruginosa* ATCC_27853_ was used as a control strain for the susceptibility testing.

### 2.4. Detection of MBLs Producing Isolates by Double Disk Synergy Test (DDST) Method

Imipenem resistance isolates were investigated for MBLs producing strains by DDST method. The bacterial suspension with turbidity equivalent to 0.5 McFarland standard was prepared and cultured on Mueller-Hinton agar [13]. For preparation of IMP-EDTA disk, 750 *μ*g of EDTA solution was added to 10 *μ*g imipenem disk and dried in an incubator [13]. At first, the bacterial suspension with turbidity equivalent to 0.5 McFarland was prepared and cultured with sterile swab on MH agar. Then, two 10 *μ*g imipenem and imipenem-EDTA disks were placed on the agar surface. After 18 hours of incubation at 35°C, the inhibition zone of imipenem disk and IMP-EDTA were measured. An increase of seven mm or more in the zone diameter for IMP-EDTA disk in comparison with imipenem disk alone was considered as a MBLs producing isolate [14].

### 2.5. DNA Extraction

DNA from* P. aeruginosa* isolates was extracted by boiling method. In this method, a number of bacterial colonies were inoculated in 10 mL of LB broth and incubation at 37°C for 16 hours. 1.5 mL of the LB broth culture was centrifuged at 13,000 ×g at room temperature for 10 min. The bacterial pellet was suspended in 300 *μ*L sterile water. The cells in the suspension were lysed by heating at 100°C for 10 min and the leftover cells were removed by centrifugation at 13,000 ×g at room temperature for 10 min. The supernatant was transferred into new tubes and used as template DNA for PCR reactions. For purity assurance, the template DNA was electrophoresed on agarose gel [10].

### 2.6. PCR Reaction for Confirmation of* Pseudomonas aeruginosa* Strains (*oprL* Gene)

PCR reaction for identification of* P. aeruginosa* strains (*oprL* gene) was performed in a final volume of 25 *μ*L: PCR Buffer (10x) 2.5 *μ*L, MgCl_2_ (50 mM) 0.75 *μ*L, dNTPs (10 mM) 1 *μ*L, forward (5′ATG-GAA-ATG-CTG-AAA-TTC-GG-<C>3′) and reverse (5′CTT-CTT-CAG-CTC-GAC-GCG-AC-<G>3′) primers [11] 500 bp (10 pmol/*μ*L) 1 *μ*L + 1 *μ*L, Taq DNA polymerase (5 U/*μ*L) 1 *μ*L, distilled water 16.75 *μ*L, and Template DNA 1 *μ*L. The thermocycler program for* oprL* gene consisted of 3 min initial denaturation at 94°C, 35 cycles of denaturation at 94°C for 1 min, annealing at 60°C for 1 min, extension at 72°C for 1 min, and final extension at 72°C for 5 min.

### 2.7. PCR Assays for Detection of MBLs Genes

#### 2.7.1. Primers

For design of primers, the nucleotide sequences of* bla-*_*VIM*_ and* bla-*_*IMP*_ genes in* P. aeruginosa* were obtained from Gene bank and aligned with Clastalw2 software (alignment program). After identification of commonality region, Gene Runner program was used for primer design. Finally for confirmation of primer specificity, they were subjected to BLAST program.

PCR reactions for* bla-*_*IMP*_ and* bla-*_*VIM*_ genes were performed in a final volume of 25 *μ*L containing the following.

*bla-*_*IMP*_. PCR Buffer (10x) 2.5 *μ*L, MgCl_2_ (50 mM) 1 *μ*L, dNTPs (10 mM) 1 *μ*L, forward (5′GTTTGAAGAAGTTAACGGGTGG3′) and reverse (5′ATAATTTGGCGGACTTTGGC3′) primers (designed) 459 bp (10 pmol/*μ*L) 1 + 1 *μ*L, Taq DNA polymerase (5 U/*μ*L) 1 *μ*L, template DNA 3 *μ*L, and distilled water 14.5 *μ*L. The thermocycler program for* bla-*_*IMP*_ gene consisted of 4 min initial denaturation at 94°C, 35 cycles of denaturation at 94°C for 1 min, annealing at 61°C for 1 min, extension at 72°C for 1 min, and final extension at 72°C for 5 min.

*bla-*_*VIM*_. PCR Buffer (10x) 2.5 *μ*L, MgCl_2_ (50 mM) 1 *μ*L, dNTPs (10 mM) 1 *μ*L, forward (5′TGGTGTTTGGTCGCATATCG3′**)** and reverse (5′GAGCAAGTCTAGACCGCCCG3′) primers (designed) 595 bp (10 pmol/*μ*L) 1 + 1 *μ*L, Taq DNA polymerase (50 U/*μ*L) 1 *μ*L, template DNA 2 *μ*L, and distilled water 15.5 *μ*L. Scheduled program for* bla-*_*VIM*_ gene by thermocycler was 4 min initial denaturation at 94°C, 35 cycles of denaturation at 94°C for 1 min, annealing at 62°C for 1 min, extension at 72°C for 1 min, and final extension at 72°C for 10 min.

Water was used as negative control and* P. aeruginosa* strains producing MBLs genes (*bla-*_*VIM*_ and* bla-*_*IMP*_) (provided from Pasteur Institute, Iran) were used as positive controls for MBL detection.

The PCR products were confirmed by gel electrophoresis in 1% (w/v) agarose gel (HT bioscience, UK) in TBE buffer and visualized with ethidium bromide staining and photographed with UV waves through Gel Documentation (Technogen, Iran) (Figures 2, 3, and 4).

## 3. Results

In total, 212* P. aeruginosa *isolates were collected. After performing initial bacteriological tests, they were confirmed to be* P. aeruginosa* by PCR assay. They were obtained from clinical specimens such as wound (*n* = 78), urine (*n* = 62), blood (*n* = 39), trachea (*n* = 16), sputum (*n* = 7), pleural fluid (*n* = 5), eye (*n* = 2), catheter (*n* = 2), and larynges (*n* = 1). The majority were from patients in burn unit (*n* = 58) and the least were from patients in cardiac unit (*n* = 1).

### 3.1. Antibiotic Susceptibility

**Figure 1:**
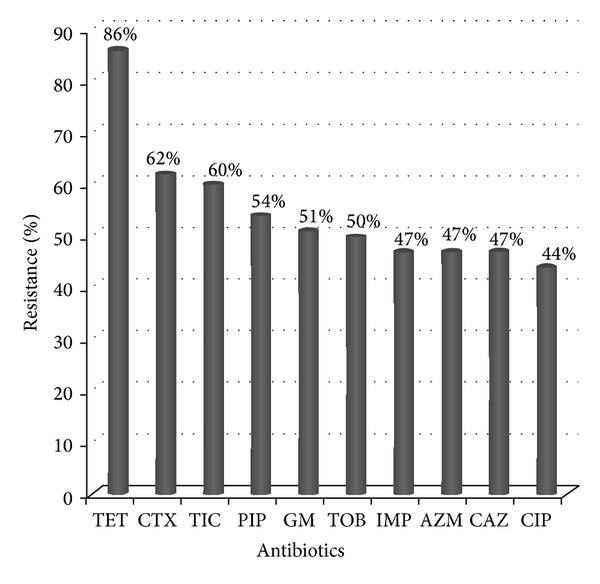
Antibiotic resistance among isolates of* P. aeruginosa.*

**Figure 2:**
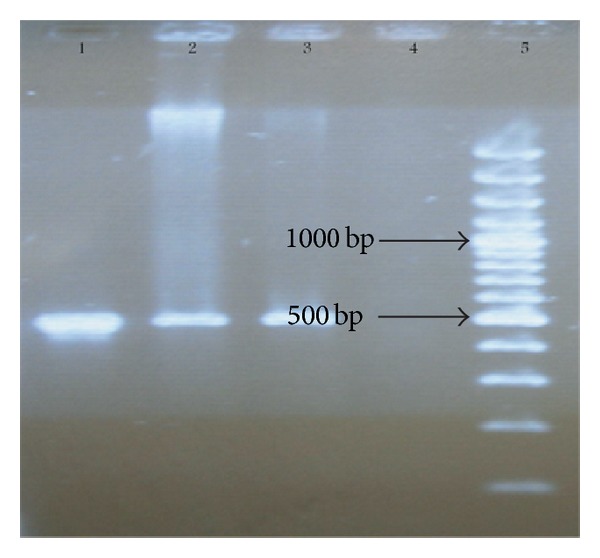
Electrophoresis of* oprL* (500 bp) PCR products on agarose gel. Line 1 shows the positive control. Lines 2 and 3 show* P. aeruginosa* strains. Line 4 shows the negative control. Line 5 shows 100–1000 bp ladder.

**Figure 3:**
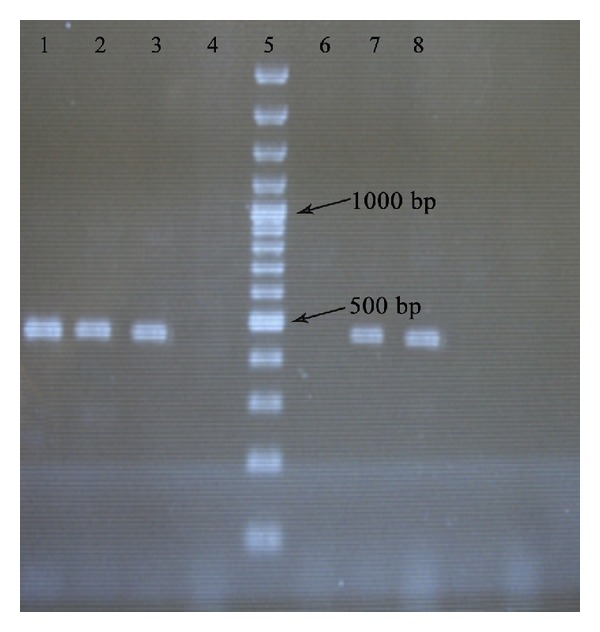
Electrophoresis of* bla-*_*IMP*_ (459 bp) PCR products on agarose gel. Line 1 is the positive control. Lines 2 and 3 show isolates positive for* IMP*. Line 4 is negative in PCR products. Line 5 shows 100–1000 bp ladder. Line 6 is negative control. Lines 7 and 8 show isolates positive for* IMP.*

**Figure 4:**
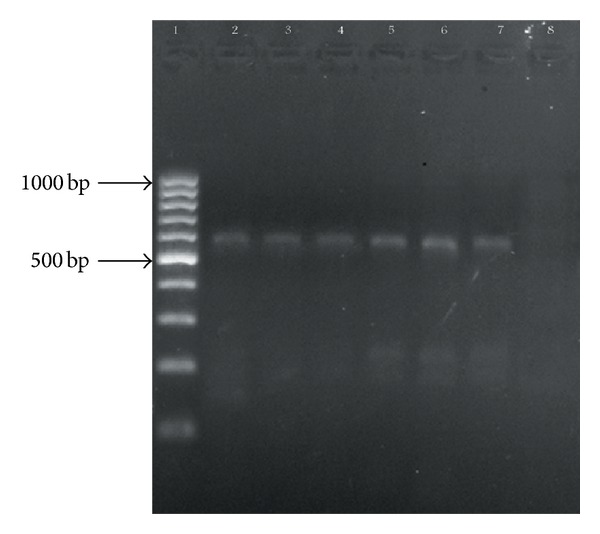
Agarose gel electrophoresis of* bla-*_*VIM*_ (595 bp) PCR products. Line 1 is 100–1000 bp ladder. Line 2 is positive control. Lines 3, 4, 5, 6, and 7 show isolates positive for* VIM*. Line 8 is negative control.

Antibiotic susceptibility of the 212 isolates in the initial disk diffusion method against 10 antibiotics is presented in Figure 1. The isolates showed high resistance to tetracycline (86%) and the most effective antibiotic was ciprofloxacin (44%).

### 3.2. MIC

Determination of MIC for imipenem by agar dilution method indicated that 47.16% (*n* = 100) of the strains were resistant to imipenem (MIC ≥ 16 *μ*g/mL).

### 3.3. Detection of MBLs Producing Isolates by Double Disk Method

In the double disk method performed on the 100 imipenem resistance isolates, 70 strains were shown to be positive by this phenotypic method.

### 3.4. Molecular Analysis

The PCR assays indicated that 20 (9%) of these strains contained the* IMP* gene, whereas 70 (33%) of them harbored the* VIM* gene.

## 4. Discussion

*Pseudomonas aeruginosa* is an opportunistic human pathogen [15, 16]. Different antibiotics are commonly used for the treatment of* Pseudomonas* infections, such as aminoglycosides, beta-lactamases, and quinolones [16–18]. Carbapenems are potent beta-lactam antibiotics against MBLs producing and multidrug resistance* P. aeruginosa* [19]. There have been many recent reports that clinical isolates of* P. aeruginosa* and Gram-negative bacilli are becoming resistant to carbapenems in many countries [19]. In recent years, MBLs have been identified from clinical isolates with increasing frequency across the world and strains that produce these enzymes have been responsible for prolonged treatment and acute infections [20]. A study from Japan showed that patients infected with MBLs producing* P. aeruginosa* needed to receive multiple antibiotics and infections leading to death due to* IMP *producing* P. aeruginosa* were more common than those with* bla-*_*IMP*_ negative* P. aeruginosa* [21]. MBLs producing* P. aeruginosa* is a serious intimidation in hospital locations especially in burn units. These strains can create significant problem in treatment and spread of resistance among other bacteria [22]. Resistance to carbapenems via acquirement of MBLs genes among* P. aeruginosa* strains have increased rapidly in Asia, Europe, and South America. This has led to a drastic change in the pattern of antibiotics usage against multidrug resistant* P. aeruginosa* [23]. Detection of MBLs producing strains can be effective for optimal treatment of patients particularly in burned and hospitalized patients and control the spread of resistance [6]. Resistance to imipenem is increasing in Iran in recent years [9, 22, 24–27]. The differences in the reported values between the present study and those reported earlier may be due to the difference in geographical regions, difference in kind of infections, the enormous usage of antibiotics, or difference in antibiotic therapy regimens in the selective hospitals in this study than those in other studies. Among the 100 isolates which were resistant to imipenem, 70 (70%) were found to be MBLs producers. In the other strains which were resistant to imipenem but were MBLs negative, resistance to imipenem may be due to efflux systems, decreased outer membrane permeability, or production of Ampc enzymes. For confirmation of MBLs producing strains, PCR is an important and accurate method [10]. In this study all isolates were screened for* VIM* and* IMP* genes by PCR. 20 isolates had* IMP* gene and 70 isolates had* VIM* gene. Of the 11 isolates that were negative with phenotypic method, 4 harbored* IMP* gene and 7 isolates had* VIM* gene as detected by PCR. Also, 3 isolates that were positive with DDST method were negative with PCR. This shows that there may be genes other than* VIM* and* IMP* responsible for MBLs trait.

Yazdi et al. isolated 126* P. aeruginosa* strains from nonburn patients in Iran in 2007. Production of MBL in these isolates was determined by* E*-test, followed by PCR to detect* bla-*_*IMP*_ and* bla-*_*VIM*_. Among 70 imipenem resistant* P. aeruginosa* strains, 8 strains produced MBLs by* E*-test all of which carried* bla-*_*VIM*_. None of them were carriers of* bla-*_*IMP*_ gene [24]. In another study in Iran during 2008, Shahcheraghi et al. collected 243* P. aeruginosa* strains from nonburn patients. 22 strains were MBLs positive; 15 of them had* bla-*_*VIM*_ and none was* bla-*_*IMP*_ positive [25]. In a study carried out in India between 2005 and 2007, among 61* P. aeruginosa* strains collected, 20 strains produced MBLs. Of the 20 MBLs confirmed strain by* E*-test, 17 strains were subjected to PCR testing. 15 of these strains were* bla-*_*VIM*_ positive and two isolates were negative for both* bla-*_*VIM*_ and* bla-*_*IMP*_ and all were negative for* bla-*_*IMP*_ [28]. In 2008, Khosravi and Mihani collected 100* P. aeruginosa* in Iran. Production of MBLs was determined both by* E*-test and PCR method. Among 41 imipenem resistant* P. aeruginosa*, 8 strains were shown to be MBLs producer by* E*-test and all of these 8 strains carried* bla-*_*VIM*_ and none of them had* bla-*_*IMP*_ [22]. In another study in Turkey, 100* P. aeruginosa* strains were collected from patients in a Turkish university hospital. One (1%) isolate was found to carry* bla-*_*VIM*_ gene, whereas 9 (9%) carried* bla-*_*IMP*_ gene. Among 9 isolates that carried* bla-*_*IMP*_ gene, only 4 isolates were shown to be MBL producer by* E*-test [29]. In our study, the percent of strains that carried* bla-*_*VIM*_ and* bla-*_*IMP*_ genes was higher than those reported in previous studies. The reasons maybe an overall increase in the extent of acquirement of MBLs genes among* P. aeruginosa.* More MBLs genes are found to be located on the class I integron and can therefore easily transfer between* P. aeruginosa* strains [7]. In the majority of studies in Iran and other countries* vim*-type MBL was the most prevalent gene reported [30–32].

## 5. Conclusion

This study illustrated that the majority of* P. aeruginosa* strains were resistant to various antibiotics. The high rate of antibiotic resistanceamong* P. aeruginosa* strains is very alarming and can be responsible for serious infections. So identification of MBLs producing strains and taking efforts to reduce the rate of transfer between different strains are important goal for treatment of* P. aeruginosa* infections.
